# Immunoglobulins in recurrent pregnancy loss: Emerging biomarkers and therapeutic targets

**DOI:** 10.1007/s11033-026-12514-2

**Published:** 2026-07-31

**Authors:** Umme Kulsum, K. S. Praveen Kumar, Pallavi Shekar, C. Kamala, Naidu M. Swati Krishnaswami, Ananya Hegde, Lakshitha V. Jayakrishna, Meghana Hangala Shivakumaraswamy, Akila Prashant

**Affiliations:** 1 Department of Medical Genetics, JSS Medical College and Hospital, JSS Academy of Higher Education & Research, Mysuru, 570015 Karnataka India; 2 Department of Biochemistry, JSS Medical College and Hospital, JSS Academy of Higher Education & Research, Mysuru, 570015 Karnataka India; 3 Department of Obstetrics and Gynaecology, JSS Medical College and Hospital, JSS Academy of Higher Education & Research, Mysuru, 570015 Karnataka India; 4 SIG-TRRG members, JSS Medical College and Hospital, JSS Academy of Higher Education & Research, Mysuru, 570015 Karnataka India

**Keywords:** Recurrent pregnancy loss, Immunoglobulins, Antibodies, Autoantibodies, Biomarkers, Intravenous immunoglobulin, Maternal–fetal immune tolerance, Precision medicine

## Abstract

Recurrent pregnancy loss (RPL) is a multifactorial reproductive disorder that affects approximately 1–5% of couples of reproductive age and is associated with considerable emotional and clinical burden. Although several genetic, anatomical, endocrine, infectious, and thrombophilic factors have been implicated, the underlying cause remains unexplained in nearly half of all cases, highlighting the importance of immune dysregulation in disease pathogenesis. Among immune factors, immunoglobulins play pivotal roles in maintaining maternal–fetal immune homeostasis by regulating immune tolerance, complement activation, autoantibody production, and inflammatory signalling. However, dysregulated humoral immune responses may disrupt placental development and fetal tolerance, thereby contributing to pregnancy failure. This review summarises current evidence regarding the biological functions of immunoglobulins in normal pregnancy and their involvement in the pathogenesis of RPL. We discuss the clinical significance of immunoglobulin-associated biomarkers, including antiphospholipid, antinuclear, anti-thyroid, and other pregnancy-related autoantibodies, and evaluate their utility in risk stratification and diagnosis. Emerging immunoglobulin-based therapeutic strategies, particularly intravenous immunoglobulin (IVIG), are reviewed in the context of current clinical evidence and guideline recommendations. Finally, we highlight the potential of comprehensive immunoglobulin profiling, integrated with clinical and molecular biomarkers, to support precision medicine approaches for individualised diagnosis, prognosis, and therapeutic decision-making in women with RPL. Continued mechanistic and translational research will be essential to validate these biomarkers and optimise personalised immunomodulatory interventions for improving pregnancy outcomes.

**Supplementary Information:**

The online version contains supplementary material available at 10.1007/s11033-026-12514-2.

## Introduction

Recurrent pregnancy loss (RPL) is a complex reproductive disorder with multifactorial etiology, posing challenges in diagnosis, treatment, and counselling [1, 2]. Even though research in reproductive genetics, obstetrics, and assisted reproduction has led to significant advances in knowledge and technology, the specific cause of RPL remains unknown for about half of all couples [3, 4]. It is important to note that traditional testing approaches, which evaluate anatomical, genetic, endocrine, and thrombotic factors as potential causes of RPL, account for only about half of cases, highlighting significant gaps in current understanding [4, 5].Pregnancy requires tightly regulated immune adaptation to maintain tolerance toward the semi-allogeneic fetus while preserving host defense [6, 7]. The fetus expresses paternal antigens; thus, it is regarded as semi-allogeneic to its mother from a purely immunological standpoint [8]. Successful pregnancy requires a finely regulated balance between immune tolerance and controlled immune activation at the maternal-fetal interface. While immune tolerance prevents maternal rejection of the semi-allogeneic fetus, controlled immune activation is equally important for embryo implantation, trophoblast invasion, spiral artery remodeling, tissue repair, and protection against invading pathogens. These tightly coordinated immune responses, mediated through interactions among immune cells, cytokines, and humoral components such as immunoglobulins, are essential for establishing and maintaining a healthy pregnancy. Dysregulation of either immune tolerance or immune activation may disrupt placental development and contribute to recurrent pregnancy loss (RPL) [6, 9].Immune dysregulation is increasingly recognized as an important contributor to unexplained RPL [10, 11]. Among the many immune components, immunoglobulins have a critical role in antigen recognition, regulating inflammation, and maintaining immune tolerance during pregnancy [7, 12]. Altered immunoglobulin profiles and pathogenic autoantibodies have been associated with immune-mediated RPL and may contribute to impaired immune tolerance, inflammation, and abnormal placentation [6, 13].These findings have prompted interest in immunoglobulin-targeted therapeutic strategies [13, 14]. IVIG exerts immunomodulatory activity by neutralizing autoantibodies, modulating cytokine production, and enhancing regulatory T-cell responses [15, 16]. Although IVIG has been found beneficial in selected cases of immune-mediated RPL, the efficacy remains variable and is still being debated [17, 18].This review focuses on immunoglobulins as central mediators of RPL pathogenesis, highlighting their roles in immune dysregulation, biomarker development, and therapeutic targeting. Furthermore, it integrates current evidence on immunoglobulin-mediated pathogenic mechanisms with emerging diagnostic and therapeutic strategies, and discusses how these insights can inform patient stratification and precision-based clinical management.

### Definition and clinical burden of recurrent pregnancy loss

Recurrent pregnancy loss (RPL) is defined as two or more failed clinical pregnancies, although the exact definition varies among professional societies. The American Society for Reproductive Medicine (ASRM) recommends evaluation after two or more failed clinical pregnancies confirmed by ultrasonography or histopathology, whereas the European Society of Human Reproduction and Embryology (ESHRE) defines RPL as the loss of two or more pregnancies before 24 weeks of gestation, irrespective of whether the losses are consecutive [2, 23, 27].

RPL affects approximately 1–2% of women of reproductive age worldwide and represents a significant reproductive health challenge because of its multifactorial etiology and substantial emotional, social, and economic burden [20, 21, 27]. Women experiencing recurrent miscarriages frequently report anxiety, depression, grief, and reduced quality of life, highlighting the need for comprehensive clinical management that addresses both medical and psychological aspects [22, 27].

Despite considerable advances in reproductive medicine, approximately 50% of RPL cases remain unexplained following routine evaluation, indicating that currently recognised etiological factors do not fully account for disease pathogenesis [3, 4, 5, 23, 28]. This persistent diagnostic gap has prompted increasing interest in identifying additional molecular and immunological mechanisms that may improve patient stratification, biomarker discovery, and the development of targeted therapeutic strategies [25, 27].

### Limitations of conventional etiological classification

Traditionally, recurrent pregnancy loss (RPL) has been classified into major etiological categories, including genetic, anatomical, endocrine, thrombotic, infectious, and immunological factors 24.Although this framework provides a systematic approach for clinical evaluation, not all identified abnormalities have a proven causal relationship with pregnancy loss, and many are based on observational associations rather than definitive mechanistic evidence [23, 26, 27, 28]. Consequently, establishing whether an identified abnormality is directly responsible for pregnancy loss remains challenging in many patients.

Current clinical practice guidelines reflect these limitations. The American Society for Reproductive Medicine (ASRM) recommends evaluation for parental chromosomal abnormalities, uterine anomalies, antiphospholipid syndrome, and selected endocrine disorders, while emphasizing that many proposed immunological investigations lack sufficient evidence for routine clinical use [2, 23]. Similarly, the European Society of Human Reproduction and Embryology (ESHRE) recommends evidence-based investigations and advises against routine testing for several emerging immunological biomarkers because of limited clinical validation and inconsistent study findings [27, 28].

Despite comprehensive evaluation, approximately 50% of RPL cases remain unexplained, highlighting the limitations of the conventional classification system [3–5, 20, 21]. This observation suggests that additional molecular, immunological, genetic, and environmental factors may contribute to disease pathogenesis but remain incompletely understood [25, 27, 29].

Rather than replacing established etiological investigations, emerging immune-based approaches should be viewed as complementary strategies that may improve patient stratification and facilitate the identification of clinically relevant subgroups. Among these, immunoglobulin-mediated mechanisms have attracted increasing interest because of their central role in humoral immunity, maternal-fetal immune tolerance, and their potential utility as biomarkers and therapeutic targets in selected patients with immune-mediated RPL [16, 25, 39]. The major etiological categories of RPL, along with their underlying pathophysiological mechanisms and key limitations, are summarised in Supplementary Table S1.

### Emerging role of immune dysregulation in RPL

Successful pregnancy depends on finely regulated interactions between the innate and adaptive immune systems that promote maternal tolerance toward the semi-allogeneic fetus while preserving protective immune responses against pathogens [8, 35, 36]. Increasing evidence indicates that disruption of this immunological equilibrium contributes to the pathogenesis of recurrent pregnancy loss (RPL), particularly in women without an identifiable anatomical, genetic, endocrine, or thrombotic cause [23, 25, 27]. Women with RPL frequently exhibit alterations in immune cell populations, including regulatory T cells (Tregs), uterine natural killer (uNK) cells, macrophages, and regulatory B cells (Bregs), together with disturbances in cytokine networks and humoral immune responses [36–38]. These abnormalities may impair trophoblast invasion, placental vascular remodeling, and maternal-fetal immune tolerance, ultimately compromising implantation and placental development [8, 35, 38].

Among the various components of the adaptive immune system, immunoglobulins have emerged as particularly important because they represent the functional effectors of humoral immunity. Physiologically, maternal immunoglobulins—especially IgG—provide passive fetal immunity and participate in immune regulation through Fc receptor-mediated signaling and modulation of inflammatory responses. Conversely, dysregulated immunoglobulin responses, including the production of pathogenic autoantibodies such as antiphospholipid antibodies (aPL), antinuclear antibodies (ANA), and anti-thyroid antibodies, can promote complement activation, endothelial dysfunction, trophoblast injury, and chronic inflammation at the maternal-fetal interface, thereby increasing the risk of pregnancy loss [39, 47, 53–55].

Beyond their pathogenic roles, immunoglobulins possess important translational value because they are readily measurable in peripheral blood, correlate with specific immune-mediated disease mechanisms, and represent therapeutic targets for interventions such as intravenous immunoglobulin (IVIG) therapy [16, 17, 39]. Consequently, immunoglobulins represent both mechanistic mediators of disease and promising biomarkers for patient stratification and individualized therapeutic approaches in immune-mediated RPL.

These observations provide the rationale for the present review, which focuses on the biological functions of immunoglobulins, their contribution to RPL pathogenesis, and their emerging diagnostic and therapeutic potential.

### Overview of maternal-fetal immune tolerance

Maternal-fetal immune tolerance is a highly coordinated immunological process that enables the maternal immune system to tolerate the semi-allogeneic fetus while maintaining adequate protection against infectious agents [8, 35, 40]. Rather than representing generalised immune suppression, pregnancy involves dynamic immune adaptation characterised by precisely regulated interactions among decidual immune cells, trophoblasts, cytokines, chemokines, and humoral immune components that together establish a supportive environment for fetal development [7, 35, 41].

Successful implantation and placental development depend on coordinated communication between trophoblast cells and maternal immune cells, including uterine natural killer (uNK) cells, regulatory T cells (Tregs), macrophages, dendritic cells, and regulatory B cells (Bregs) [42, 43, 46]. These immune cells regulate trophoblast invasion, spiral artery remodelling, angiogenesis, and local immune tolerance by producing anti-inflammatory cytokines such as IL-10 and TGF-β, while simultaneously preserving the capacity to respond to microbial pathogens [38, 40, 42].

Disruption of these tightly regulated immune interactions may impair trophoblast invasion, placental vascular remodelling, and fetal immune tolerance, thereby contributing to recurrent pregnancy loss [36, 39]. Increasing evidence indicates that abnormalities in both cellular and humoral immunity—including dysregulated immunoglobulin responses—may disturb this immunological equilibrium and promote inflammatory and antibody-mediated mechanisms that compromise pregnancy [25, 39, 46].

**Fig. 1:**
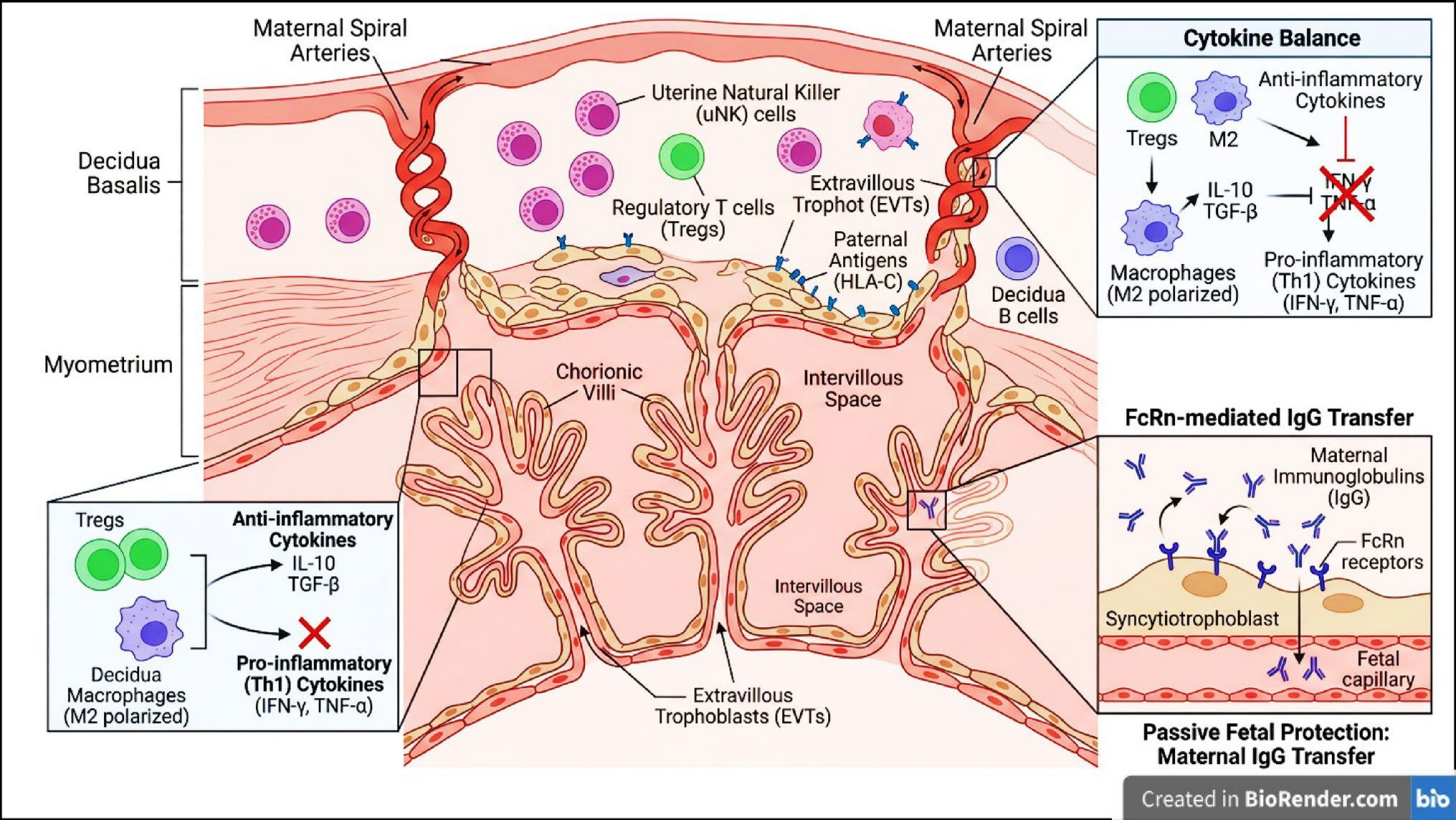
Maternal-fetal immune tolerance at the implantation site

A schematic overview of the principal immune cell interactions that establish maternal-fetal immune tolerance during early pregnancy is presented in Fig. 1. Although these pathways are supported by experimental and clinical evidence, their precise contribution to individual cases of RPL remains an active area of investigation.

This figure presents a cross-sectional view of the maternal-fetal interface at the beginning of implantation, including the maternal decidua containing key immune cells (uNK cells, Tregs, etc.), trophoblast invasion of paternal antigens, B cells, macrophages, spiral arteries, and placental transfer of IgG via FcRn. Arrows indicate anti-inflammatory cytokines (IL-10, TGF-β) that promote tolerance and repress Th1 pro-inflammatory reactions.

## Immunological adaptations during normal pregnancy

**Fig. 2:**
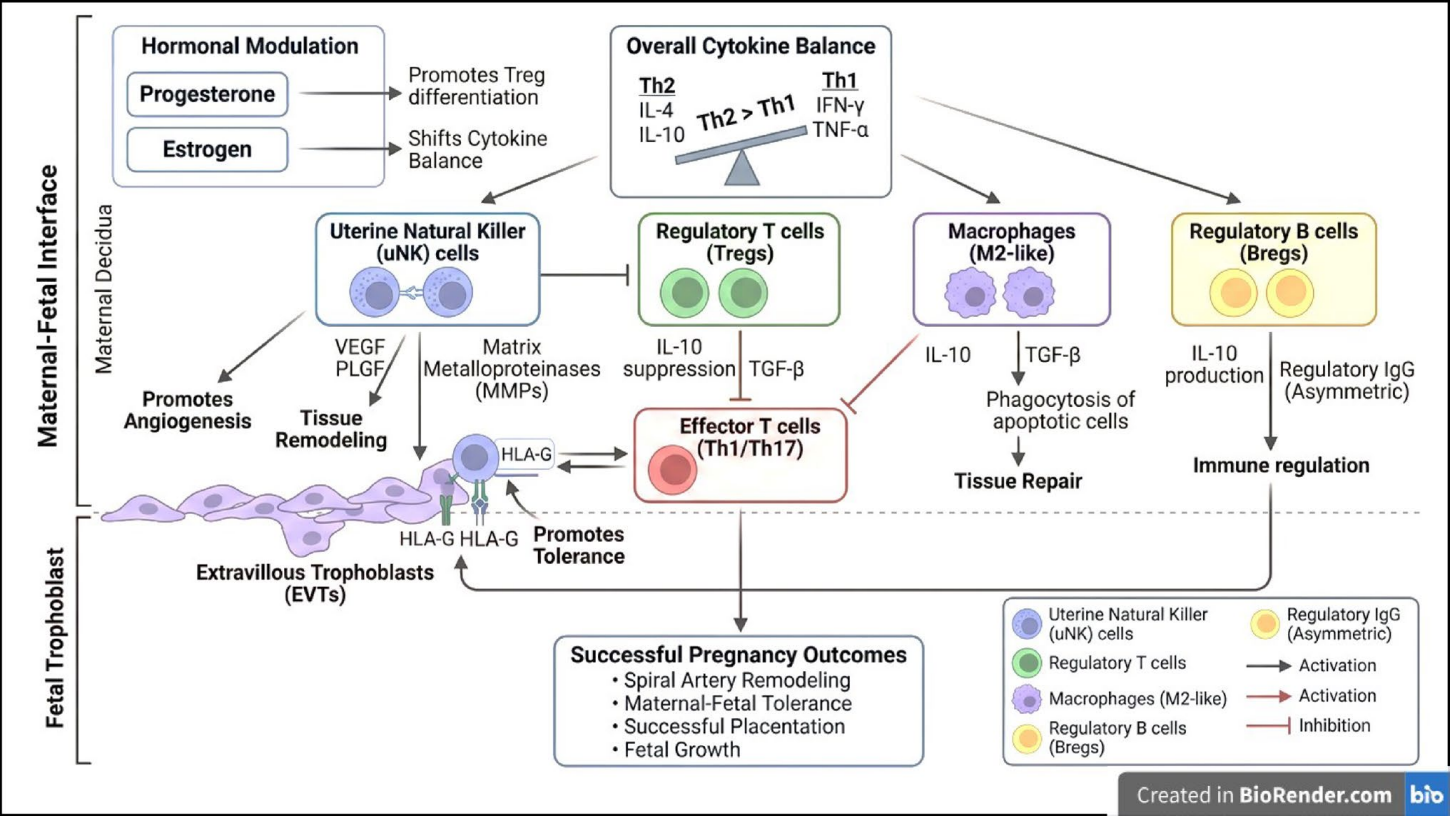
Immune cell interactions during normal pregnancy adaptations

The immune changes during pregnancy represent a dynamic and tightly regulated process which enables maternal tolerance toward the fetus while supporting successful placentation [8, 28]. In early pregnancy, the maternal immune system is transitioning into a primarily anti-inflammatory or tolerogenic state, which will allow for the implantation of the embryo [33]. This transition does not reflect total immune suppression; there is still a selective change to the innate and adaptive immune response during this transition [34]. During this time, the decidual immune cells in the uterus, which include uterine natural killer (uNK) cells, macrophages and dendritic cells, execute important functions that facilitate angiogenesis (formation of new blood vessels), tissue remodelling, and immune regulation [35]. Overall, the Th2 immune responses are dominant, and this is facilitated by the presence of greater numbers of regulatory immune cell populations [29]. In addition to the immune changes during pregnancy, the increased levels of hormones such as progesterone and estrogen can also have profound effects on the immune system [38]. Disruption of these adaptations may contribute to adverse pregnancy outcomes [22]. Figure 2 shows the coordinated interaction of decidual immune cells and cytokines during normal pregnancy. As shown in Fig. 2, multiple immune pathways act in a coordinated manner to maintain pregnancy; however, the relative contribution of each pathway in the context of RPL remains incompletely understood.

The following flowchart shows the important immunological adaptations at the maternal-fetal interface, depicting communication between uterine NK cells (uNK), regulatory T cells (Tregs), macrophages, and trophoblast-bearing B cells. Directional arrows depict mechanisms such as IL-10/TGF-β suppression by Tregs, uNK-driven vascular remodelling, and Th2 cytokine dominance influenced by progesterone, leading to successful placentation.

### Role of B cells, immunoglobulins, NK Cells, Tregs, and cytokines

There are many immunological components working together to create maternal-fetal immune tolerance [8]. B cells play an important role in pregnancy because B cells produce antibodies and cytokines as well as help regulate the immune system [39]. Immunoglobulin, especially IgG subclasses, provide passive immunity to the fetus and help regulate the immune response at the maternal-fetal interface [40]. Uterine NK cells, which are the largest subset of immune cells in the decidua, help support normal placental development through their regulation of trophoblast invasion and vascular formation rather than through their cytotoxic activity [35, 36]. T regulatory cells (Tregs) are critical for suppressing maternal immune response to fetal antigens, and maintaining immune balance between maternal and fetal antigens [33]. Success in achieving pregnancy depends on the maintenance of a balance between pro-inflammatory and anti-inflammatory cytokines throughout the pregnancy process [31]. Any disruption of any of these components can negatively impact the course of pregnancy [29].

### Breakdown of immune tolerance in recurrent pregnancy loss

The maternal-fetal immune tolerance mechanisms are often disrupted in cases of RPL [22]. Women with RPL often have pro-inflammatory immune responses and altered immune cell types along with altered cytokine production [30, 31]. Abnormal activation of uterine NK cells can inhibit the ability of trophoblasts to invade and the forming of placental vascularization [35]. Similarly, a deficiency or dysfunction of regulatory T cell (Tregs) impairs immune tolerance maintenance [33]. Dysregulation in B cell function and abnormal production of immunoglobulins, including pathogenic autoantibodies, can lead to an imbalance in the immune system and may directly damage the maternal-fetal interface [39]. The combination of these molecular abnormalities creates a local environment that is harmful to implantation and may be a factor in pregnancy loss [32]. In order to identify immunological targets and develop reliable therapeutic strategies in RPL, understanding the mechanisms that lead to tolerance breakdown is important [37].

## Immunoglobulins: structure, classes, and functional diversity

### Overview of immunoglobulin classes (IgG, IgM, IgA, IgE)

**Fig. 3:**
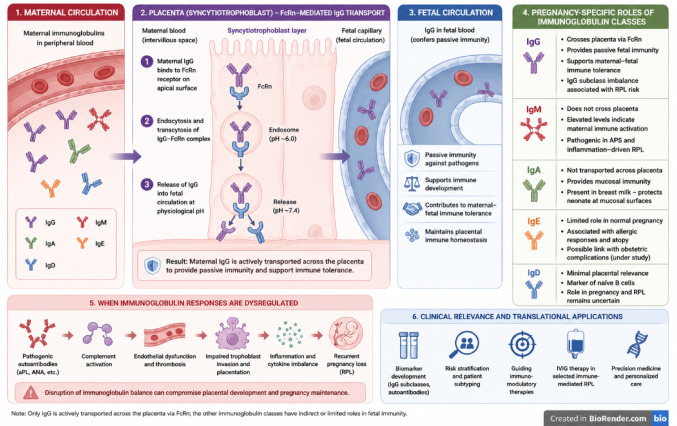
Pregnancy-specific functions of immunoglobulins, FcRn-mediated placental transport, and their role in recurrent pregnancy loss

Immunoglobulins are glycoproteins produced by B lymphocytes and plasma cells in response to antigen that are critical components of humoral immunity [41]. Humoral immunity consists of four different isotypes of immunoglobulins: IgG, IgM, IgA and IgE; each of these classes of immunoglobulins have unique properties and functions [41]. IgM, as the primary antibody of early immune response, contributes to complement activation, which may be relevant in inflammatory mechanisms associated with RPL [42]. IgA is the immunoglobulin responsible for mediating mucosal immunity, while IgE is the immunoglobulin associated with allergy and hypersensitivity [43]. IgG is the most abundant circulating immunoglobulin, and its ability to cross the placenta makes it particularly relevant in the context of maternal–fetal immune regulation [40]. IgG-mediated regulation is critical for both maternal-fetal immune tolerance and the immunological protection from infectious agents for the fetus as it develops [44]. Perturbations in immunoglobulin levels or function have been associated with immune dysregulation and complications during pregnancy, including RPL [32]. The structural characteristics of the four major classes of immunoglobulins, including the Y-shaped structure of IgG along with its Fab and Fc domains, subclass variation of IgG, pentameric forms of IgM, and the mechanism for placental IgG transfer through the neonatal Fc receptor (FcRn) is illustrated in Fig. 3. While this figure highlights the structural diversity and functional roles of immunoglobulins, the extent to which these structural differences translate into clinically relevant effects in RPL requires further investigation.

Schematic illustration of the major immunoglobulin classes and subclasses with emphasis on their roles during pregnancy. The figure highlights FcRn-mediated transplacental transport of maternal IgG, passive fetal immunity, maintenance of maternal-fetal immune tolerance, and the contribution of pathogenic autoantibodies to recurrent pregnancy loss. Pregnancy-specific biological functions and potential clinical applications, including biomarker development and immunoglobulin-based therapies, are also summarized.

### IgG subclasses and placental transfer

There are four IgG subclasses, which are IgG1, IgG2, IgG3, and IgG4, that contain unique structures in their Fc regions in addition to different modes of working in our immune system. These functional differences are clinically important, as they influence complement activation and Fc receptor binding, which may contribute to differential pregnancy outcomes in RPL. The maternal IgG that crosses through the placenta to the fetus is mediated by the neonatal Fc receptor (FcRn); the transfer efficiency of IgG1 and IgG3 is greater than that of IgG2 and IgG4 during the transport of maternal IgG to the fetus [44]. Disruption of IgG subclass distributions may affect both fetal immune protection and immune tolerance in the maternal-fetal interface [40].

### Functional relevance of immunoglobulins at the maternal-fetal interface

At the maternal-fetal interface, immunoglobulins have several immune functions including regulation of immunity, the defence against pathogens and the establishment of tolerance [8]. Protective IgG antibodies play an important role in maintaining the integrity of the placenta by neutralizing infectious challenges and preventing excessive inflammatory responses [40]. Immunoglobulins modulate the activity of immune cells as well as their production of cytokines and thus promote a tolerogenic environment for immunity [16]. Conversely, pathogenic autoantibodies may also inhibit trophoblastic invasion, alter placental function and trigger inflammatory cascades [32]. The relative balance of protective and pathogenic immunoglobulin response thus has fundamental importance in pregnancy outcome and a contributing factor to the development of RPL [17]. The functional roles of major immunoglobulin classes in normal pregnancy and their mechanistic contributions to RPL, along with their clinical relevance, are summarized in Table 1.

**Table 1:** Functional roles of immunoglobulins in pregnancy and their association with recurrent pregnancy loss

Immunoglobulin Type	Physiological role in pregnancy	Mechanistic contribution to RPL	Clinical relevance
IgG [40]	Mediates placental transfer via FcRn; provides passive fetal immunity; regulates immune responses	Autoantibody-mediated complement activation, endothelial dysfunction, and impaired trophoblast invasion	Key biomarker; IgG subclass profiling (IgG1–IgG4) may aid in risk stratification
IgM [45]	Primary immune response; complement activation	Elevated levels associated with autoimmune activation and localized inflammation	Indicator of active immune dysregulation
IgA [43]	Supports mucosal immunity and immune modulation	Altered signalling at the maternal-fetal interface may contribute to immune balance	Limited but emerging clinical relevance
Autoantibodies [32]	Normally absent or present at low, non-pathogenic levels	Target phospholipids, nuclear antigens, or placental proteins, leading to immune-mediated tissue damage	Strong diagnostic and prognostic significance in RPL

## Immunoglobulins as diagnostic and prognostic biomarkers in RPL

Immunoglobulin (Ig) based biomarkers are important for the diagnosis and characterization of immune-mediated pathways involved in RPL [32]. Pathogenic autoantibodies can directly affect processes that are vital to implantation, placentation, and fetal survival [29]. Of these, antiphospholipid and antinuclear autoantibodies have been researched most thoroughly and established as clinically relevant [46]. Their identification can assist with diagnosis and risk analysis and influence the decision for treatment. Additional emerging autoantibodies have attracted attention for explaining some cases of unexplained RPL [27]. However, there are still significant challenges related to assay standardization, inter-laboratory variability, and clinical interpretation; thus, using an integrated biomarker panel rather than relying on individual markers in isolation is necessary [46]. Table 2 summarizes key immunoglobulin-related biomarkers in RPL, along with their biological targets, pathogenic mechanisms, and clinical relevance.

Although several biomarkers have been implicated in RPL, their clinical utility remains variable due to differences in sensitivity, specificity, and study methodologies, underscoring the need for further validation.

**Table 2:** Immunoglobulin-based biomarkers in recurrent pregnancy loss: Biological roles and clinical implications

Biomarker	Target / Function	Pathogenic Mechanism	Clinical Utility
Antiphospholipid antibodies (aPL) [46]	Phospholipid-binding proteins (β2-GPI, cardiolipin)	Induce thrombosis, complement activation, impaired trophoblast invasion	Established diagnostic marker for APS; guides anticoagulant therapy
Antinuclear antibodies (ANA) [47]	Nuclear antigens	Reflects systemic immune dysregulation and altered immune tolerance	Associated with increased miscarriage risk; low disease specificity
Anti-thyroid antibodies (TPO, TgAb) [48]	Thyroid antigens	Immune-mediated effects independent of thyroid function	Predictor of miscarriage even in euthyroid women
Anti-prothrombin / anti-protein S antibodies [49]	Coagulation factors	Prothrombotic state affecting placental perfusion	Emerging biomarkers requiring further validation
Anti-sperm antibodies [50]	Sperm antigens	Interfere with fertilization and early embryonic development	Limited current clinical application

### Antiphospholipid antibodies (aPL)

Antiphospholipid antibodies have been recognized as the most well-established immunological markers associated with RPL and are required for the diagnosis of antiphospholipid syndrome (APS) [46]. The antiphospholipid antibody collection includes three markers: lupus anticoagulant, anticardiolipin antibodies, and anti-β2 glycoprotein I antibodies of both the IgG and IgM isotypes [51]. Antiphospholipid antibodies produce an increased risk of pregnancy loss via multiple mechanisms, including placental thrombosis, insufficient trophoblast invasion, and complement activation [52]. Lupus anticoagulant is presently the most predictive marker for pregnancy losses among the antiphospholipid antibody markers. Persistent positivity for antiphospholipid antibodies is of considerable diagnostic and prognostic value and impacts clinical management, including the decision-making for anticoagulant therapy and immunomodulatory therapy [53].

### Antinuclear antibodies (ANA)

Women with RPL may have antinuclear antibodies in their blood even if they are not suffering from systemic autoimmune disease. This may reflect underlying immune dysregulation; however, the clinical relevance remains uncertain due to high background positivity in healthy individuals [54]. Recent research has shown that both an elevated ANA level and a specific profile of immunofluorescence have been associated with an increased chance of miscarriage (RPL) [55]. Nevertheless, because there is so much variation between ANA and its clinical significance, ANA are often positive at low titers in healthy women, as well. Thus, when interpreting ANA, it is generally important to consider other tests that measure both immunologic parameters and clinical features as opposed to only using ANA as the sole means of diagnosis [55, 56].

### Emerging autoantibody biomarkers

Building on the immunological mechanisms described earlier, emerging autoantibody biomarkers reflect underlying immune dysregulation and may assist in identifying patients at increased risk of RPL. Several emerging serological markers have been studied in RPL beyond traditional autoantibodies. Examples of these include anti-prothrombin and anti-protein S antibody manifestations, both of which can create a propensity toward clotting that causes a decrease in blood flow to the developing fetus [57]. Autoantibodies against thyroid antigens, particularly anti-thyroid peroxidase (TPO) and anti-thyroglobulin (TgAb), have been associated with an increased risk of recurrent pregnancy loss and miscarriage, even in women with normal thyroid function (euthyroid women) [65]. This observation suggests that thyroid autoantibodies may contribute to pregnancy loss through immune-mediated mechanisms independent of overt thyroid dysfunction [58]. Although the precise mechanisms remain incompletely understood, thyroid autoantibodies may reflect underlying immune dysregulation, promote inflammatory responses at the maternal-fetal interface, or interfere with placental development, thereby increasing the risk of pregnancy loss [55, 65].

These findings highlight the potential to expand the diagnostic options available for RPL, further validation must be completed via large prospective trials before implementing any new blood test systematically into clinical practice [55]. Collectively, these emerging immunoglobulin-related biomarkers provide deeper insight into the immunopathological basis of RPL and may serve as a foundation for patient stratification strategies, particularly within the context of precision medicine and targeted therapeutic approaches discussed in subsequent sections. Having established the physiological roles of immunoglobulins during pregnancy, the following section discusses their diagnostic and prognostic significance in recurrent pregnancy loss.

## Immunoglobulin-mediated pathogenic mechanisms in recurrent pregnancy loss

**Fig. 4:**
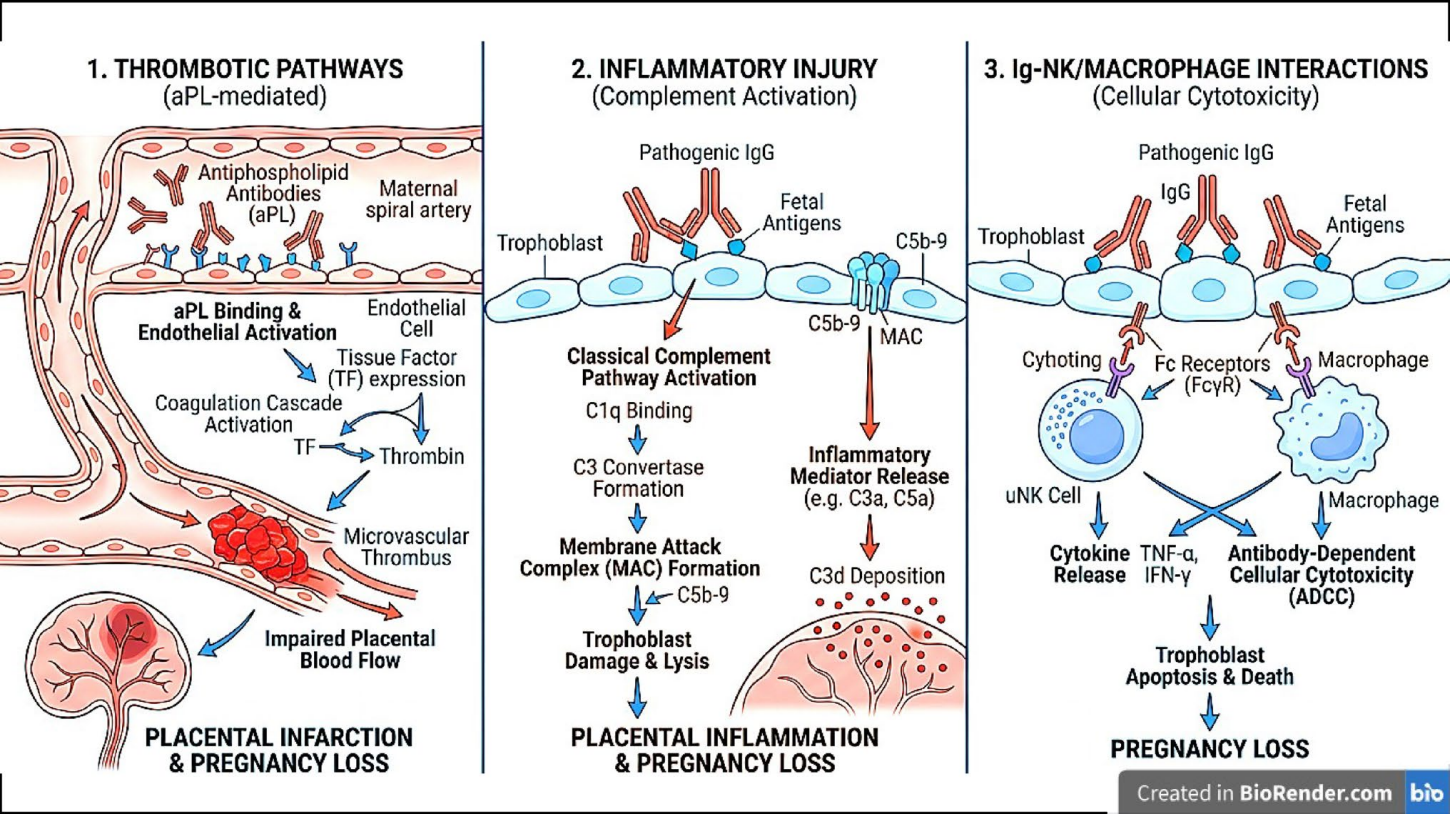
Immunoglobulin-mediated pathogenic mechanisms in recurrent pregnancy loss

Following the discussion of their clinical utility as biomarkers, it is essential to understand the molecular mechanisms through which dysregulated immunoglobulin responses contribute to recurrent pregnancy loss. Immunoglobulins serve both as indicators of immune system impairment and as direct contributors to maternal-fetal pathology. Pathogenic antibodies are capable of initiating a series of inflammatory, thrombotic, and immune-related cascades that disrupt implantation and normal placental development [29]. Through their interactions with complement proteins, endothelial cells, trophoblasts, and immune effector cells, immunoglobulins affect the processes required to maintain pregnancy. Failure of immune modulation converts physiologic processes into pathologic outcomes that ultimately lead to unsuccessful clinical pregnancy [17]. Thus, through an understanding of how these mechanisms work, we have justification for using immunomodulation to treat RPL. The potential immunoglobulin-mediated pathogenic mechanisms contributing to RPL are illustrated in Fig. 4. As shown, multiple pathways may act in parallel to disrupt trophoblast function and placental development; however, the relative contribution of each mechanism remains unclear, as much of the supporting evidence is derived from experimental and in vitro studies rather than large-scale clinical investigations.

Three-panel diagram describing: (1) Thrombotic processes involving antiphospholipid antibodies (aPL) binding to endothelial cells, triggering the coagulation cascade and microvascular thrombosis that compromises placental circulation; (2) Damage through classical complement activation (binding of C1q to pathogenic IgG, formation of C3 convertase, membrane attack complex deposition) resulting in trophoblast damage; (3) IgG-Fc receptor interaction on uterine NK cells and macrophages, which activates the release of pro-inflammatory cytokines (TNF-α, IFN-γ) and cytotoxicity toward trophoblasts. Arrows indicate progression to implantation failure and pregnancy loss.

### Complement activation and inflammatory injury

The classical complement system can be activated by pathogenic antibodies, leading to increased inflammatory damage at the maternal-fetal interface [59]. When activated, complement produces pro-inflammatory mediators and causes formation of the membrane attack complex (MAC), both of which can adversely affect the trophoblast and placental tissue [60]. A recent study reported significant increase in complement in placentas belonging to women who have had RPL, indicating that antibodies play a role in causing tissue damage through an inflammatory mechanism [61]. The resultant damage could impair fetal growth, compromise the vascular integrity of the placenta, and place the fetus at greater risk for loss [60]. Therefore, dysregulated complement activation is a major mechanism by which pathogenic antibodies increase inflammation and injury in the early stages of pregnancy [62].

### Endothelial dysfunction and thrombotic pathways

One of the most important causes of RPL is the antibody-mediated endothelial dysfunction. This is particularly evident in women with thrombophilia, where antiphospholipid and other pathogenic autoantibodies interact with endothelial cell surfaces to induce a pro-inflammatory, pro-thrombotic state [63]. This pro-inflammatory, pro-thrombotic state leads to platelet aggregation, activation of the coagulation cascade, thrombi in the microcirculation, and decreased blood flow to the placenta, all of which reduce the delivery of oxygen and other essential nutrients to the developing embryo [64]. The presence of thrombi in the microvasculature directly decreases the perfusion of the placenta, and is strongly correlated with early pregnancy loss (EPL); thus, immunoglobulins play a critical role in the pathogenesis of endothelial and vascular dysfunction in RPL [65].

### Impaired trophoblast invasion and placentation

Regulation of trophoblasts and placenta development is required for a successful pregnancy and these processes are highly dependent on interactions with the immune system [8]. Pathogenic immunoglobulins alter intracellular signalling in trophoblasts, thereby promoting apoptosis and inhibiting cell migration and invasion, which leads to trophoblast dysfunction [29]. Immunoglobulin autoantibodies that target phospholipid-binding proteins or placental antigens could hinder trophoblast differentiation and spiral artery remodelling. The result is shallow placentation and placental insufficiency which significantly increases both the risk for pregnancy loss [66]. Therefore, the ability of immunoglobulins to inhibit trophoblast invasion represents a major mechanistic contribution to impaired placental development in cases of RPL and immune aberration [32].

### Interaction of immunoglobulins with NK cells and macrophages

Naturally occurring antibodies can enhance the activation of macrophages and natural killer (NK) cells through their interactions with Fc receptors present on these cells [67]. The binding of pathogenic antibodies to the Fc receptors can enhance immune cell activation by increasing their cytotoxicity and the production of pro-inflammatory cytokines [68]. In patients with RPL, abnormal antibody-Fc receptor interactions impair the supportive functions of uterine NK cells and shift them towards a destructive phenotype [29]. Similarly, pathogenic immunoglobulins can activate macrophages, resulting in increased inflammation and tissue damage. The altered immune responses of these cells lead to the development of immune-mediated placental injury and the failure of pregnancy [8]. Collectively, these mechanistic insights provide a biological foundation for the clinical relevance of immunoglobulin-based biomarkers and support the rationale for therapeutic targeting in RPL.

## Regulatory B cells, immunoglobulin balance, and immune homeostasis

### IL-10–producing regulatory B cells (Bregs)

The pathogenic mechanisms described above are closely linked to alterations in humoral immune regulation. This section focuses on the immunological pathways that connect dysregulated immunoglobulin responses with pregnancy failure. Regulatory B cells (Bregs) are a distinct subset of B lymphocytes whose primary means of maintaining immune tolerance is by secreting interleukin-10 (IL-10) among other anti-inflammatory cytokines [69]. They foster tolerance to fetal antigens and mitigate excessive immune activation of the mother during healthy pregnancies [39]. The IL-10 produced by Bregs leads to an inhibition of release of pro-inflammatory cytokines, a reduction in antigen presentation, and an amplification of regulatory T cells [70]. Women who experience RPL have been found to have reduced numbers of Bregs and a decreased capacity for IL-10 production indicating that there is inadequate immunological regulation at the maternal-fetal interface [71]. Therefore, dysfunctional Bregs can lead to the breakdown of maternal-fetal tolerance and the continued occurrence of immune-mediated pregnancy loss [72].

### Altered IgG subclass distribution and immune imbalance

The amount of immunoglobulin produced during pregnancy is regulated by homeostasis, and changes in the levels of total IgG or the types of IgG may be linked to poor pregnancy outcomes [6]. Abnormal immunoglobulin production can lead to abnormal Fc receptor binding following immune stimulation, contributing to a pro-inflammatory immune environment. For example, increased levels of IgG1 or IgG3, together with lower levels of IgG4 (an anti-inflammatory antibody), may tip the balance of the immune system to activate complement proteins and increase effector cell activation [73]. Such imbalances can compromise maternal-fetal tolerance and impair placental development [8]. These results highlight the need for tight regulation of immunoglobulin production to maintain immune homeostasis during pregnancy [6, 74].

### Crosstalk between Bregs, Tregs, and NK Cells in RPL

The coordinated interactions between regulatory B (Breg) cells, regulatory T (Treg) cells, and uterine natural killer (uNK) cells are critical to maintain immune homeostasis at the maternal-fetal interface [6]. Bregs promote a state of tolerance via the production of IL-10 which facilitates the expansion and functioning of Tregs [75]. Tregs modulate the activity of uNK cells to limit the excessive cytotoxicity of uNK cells while supporting placental development [76]. Regulated uNK cell activity allows balanced immune regulation whereas excess activation of uNK cells results in increased production of pro-inflammatory cytokines and the potential for trophoblast damage [77]. Disruption of any component of this regulatory network through dysfunction of one or more of these immune cell populations can lead to an immune imbalance, placental failure, or RPL [29]. Therefore, disrupted communication between these subsets of immune cells contributes to the immune environment that results in immunologically-mediated RPL [6].

## Immunoglobulins as therapeutic targets in recurrent pregnancy loss

Understanding these pathogenic mechanisms provides the biological rationale for immunoglobulin-based therapeutic interventions aimed at restoring maternal–fetal immune tolerance. Interest in immunoglobulin-targeted therapies stems from their association with immune dysregulation. Immunoglobulin-mediated pathways play an important role in immune-related reproductive loss [32]. As they play a vital role in regulating the immune system, inflammation, and tolerance between the mother and fetus; targeting immunoglobulin pathways is justified as a method for providing an intervention in the treatment of immune-mediated reproductive loss [28]. Immunotherapy can counteract pathogenic antibody responses and reinforce regulatory immune pathways, therefore, a rational method of improving pregnancy outcomes for women who have experienced RPL related to immune-related mechanisms [32]. There are currently multiple options being tested as potential immunoglobulin therapies; however, one of the most researched methods of treatment so far is intravenous administration of immunoglobulin (IVIG) [17].

### Rationale for immunoglobulin-based therapies

Evidence is available that the lack of normal humoral immune reactions will cause RPL [29]. The action of pathogenic autoantibodies, an altered distribution of IgG subclasses, or a deficiency in immune regulation can damage the ability of the mother to tolerate the child and fetal development [31, 78]. Treatment with immunoglobulins attempts to restore normal immune balance by either blocking or neutralizing the effect of pathogenic antibodies and by regulating the activity of immune cells. Immunoglobulin therapies are especially effective in patients with autoimmune disordered functions and in patients with antibody-positive RPL where evidence supports a pathogenic consequence of humoral immunity on pregnancy [79]. Instead of focusing on the clinical manifestation of RPL (the result of immunological disturbances), immunoglobulin-based treatments target and treat the disturbance itself (the cause of the clinical manifestation) making their use more mechanism-based than prescription medication use for RPL [17].

### Intravenous Immunoglobulin (IVIG): mechanisms of action

Based on the immunoglobulin-mediated mechanisms outlined in previous sections, IVIG has been proposed as a therapeutic strategy to modulate immune dysfunction in RPL. IVIG exerts multiple immunomodulatory effects, including multiple pathways by which IVIG modulates the way Fc receptors are expressed [80]. By changing these molecules, IVIG reduces antibody-mediated activation of the immune system and inflammation. At the maternal-fetal interface, IVIG reduces uterine NK cell cytotoxicity and helps maintain trophoblast integrity [17]. IVIG also increases the proliferation and functionality of regulatory T cells (Tregs) and promotes IL-10 secretion from Bregs, thereby enhancing the establishment of immune tolerance at the maternal-fetal interface [81]. Additionally, IVIG may neutralize pathogenic autoantibodies and reduce complement activation. Collectively, these immunomodulatory mechanisms may help restore immune homeostasis in patients with RPL [32].

### Clinical evidence for IVIG in recurrent pregnancy loss

Clinical trials investigating IVIG for RPL have produced inconsistent results, largely due to heterogeneity in patient selection, study design, and outcome measures [82]. Some studies suggest that women with immune-mediated RPL, particularly those who have high levels of NK cell activity or autoantibody positivity, may benefit most from treatment with IVIG [17, 37]. However, it is critical to note that the current guidelines from the American Society for Reproductive Medicine (ASRM) and the European Society of Human Reproduction and Embryology (ESHRE) do not recommend routine IVIG for unexplained RPL due to insufficient high-quality evidence [19, 83].

Multiple randomized controlled trials have not identified any beneficial effects of IVIG on live births relative to placebo in RPL patients overall. In a meta-analysis published by Kofod et al., they did not find any evidence that IVIG utility in unselected populations improved the rate of live births compared to women without an immune-related cause for RPL [82]. Many studies that found positive benefits from the administration of IVIG were small or lacked placebo, or did not account for potential confounding factors. Therefore, while a subset of patients with documented immune dysfunction may benefit, IVIG should be reserved for carefully selected individuals, ideally within clinical trials or under specialist guidance. Despite these mechanistic insights and therapeutic potential, the translation of immunoglobulin-based interventions into consistent clinical benefit remains challenging, as discussed in the following section.

### Safety, timing, and dosage considerations

IVIG is generally well tolerated when given under the right conditions and with proper supervision. Most side effects are minor and temporary. Headaches, fever, and infusion-related reactions are common side effects; however, severe adverse effects are rare [84]. The peri-implantation and early pregnancy periods appear to be when the greatest benefit is observed [17]. Dosing of IVIG varies substantially from one report to another and lack of a uniform dosing protocol may explain some of the variability seen clinically. Standardizing dosages of IVIG and systematically identifying which patients will get the greatest benefit from IVIG are two significant hurdles to optimizing IVIG utilization as part of management of RPL [37]. Given that these pathogenic pathways are mediated by immunoglobulins, therapeutic strategies such as IVIG aim to modulate these same immune mechanisms.

## Precision medicine approach: patient stratification based on immunoglobulin profiles

Despite advances in biomarker discovery and immunomodulatory therapy, considerable heterogeneity exists among women with recurrent pregnancy loss. Precision medicine approaches seek to integrate immunoglobulin profiles with clinical and molecular characteristics to guide individualised management. RPL exhibits significant clinical and biological variability. Building on the biomarker profiles described earlier, precision medicine approaches aim to stratify patients based on immunological characteristics to enable more targeted and individualised therapeutic interventions. This highlights the need to establish a precision medicine framework that enables mechanism-based diagnosis and individualised therapies [13]. Comprehensive immunoglobulin profiling, including the assessment of immunoglobulin isotypes, IgG subclass distribution, autoantibody specificity (e.g., antiphospholipid antibodies, antinuclear antibodies, and anti-thyroid antibodies), antibody titers, and their persistence over time, has the potential to improve risk stratification in women with recurrent pregnancy loss (RPL) [39, 62, 93]. Such profiling may help distinguish patients with immune-mediated RPL from those with non-immunological causes, thereby facilitating more accurate diagnosis and individualised clinical management. Furthermore, integrating immunoglobulin profiles with clinical characteristics and other immunological biomarkers may help identify patients more likely to benefit from targeted immunomodulatory therapies, such as intravenous immunoglobulin (IVIG), while avoiding unnecessary treatment in women unlikely to respond [17, 39, 90]. Because RPL is a clinically heterogeneous disorder, no single immunoglobulin biomarker is sufficient for patient stratification. Instead, combining immunoglobulin profiles with clinical history, immune cell phenotyping, genetic susceptibility, and multi-omics approaches provides a more comprehensive framework for precision medicine and individualised therapeutic decision-making [35, 36].

Effective stratification requires evaluation of antibody characteristics. Such evaluations will include identification of the type of antibodies (isotype), subclass distribution of IgG among patients, quantification of antibody titer levels, and documentation of how long antibodies persist over time [85]. High levels (persistently elevated titers) of autoantibodies (antiphospholipid antibodies or anti-thyroid antibodies) may indicate immunologically mediated disease processes that are likely to respond to therapy; on the other hand, low titer or transient antibody positivity may simply indicate non-specific immune activity without any secondary clinical significance [86, 87]. In addition, IgG subclass profiling provides additional refinement to this stratification, as it elucidates the differences between Fc receptor binding, complement activation potential and immune effector cell engagement among different IgG subclasses [73].

The integration of immunoglobulin biomarker information with clinical characteristics, immune cell phenotyping, genetic susceptibility, and multi-omic assessments provides a comprehensive basis for precision classification of patients [28]. These integrated models support individualized clinical decision-making by providing guidance for the selection, timing, and intensity of immunomodulatory treatments while preventing unnecessary interventions in low-risk patients [29]. Longitudinal immunoglobulin profiling allows for the ongoing evaluation of disease activity and treatment response over time. Immunoglobulin-based stratification supports personalized management of immune-mediated RPL and enhances the quality of care [32]. Integration of biomarker profiles with these underlying mechanisms enables more precise patient stratification and targeted therapy selection.

## Discussion

This review summarizes the significant immunoglobulin-related pathways hypothesized to play a role in RPL [29]. Although accumulating evidence implicates immunoglobulin-mediated immune pathways in RPL, translating these mechanistic insights into consistent clinical applications remains challenging.

The relationship between autoantibody levels, particularly antiphospholipid and antinuclear antibodies, and clinical outcomes has been described; however, due to variable diagnostic specificity and outcome prediction across studies, these associations should be interpreted cautiously [66]. Several factors may contribute to this variability, including study design, patient selection (e.g., APS) and the laboratory methods used for antibody detection [13].

One of the major challenges in the current literature is the significant heterogeneity across studies, including differences in diagnostic criteria, patient populations, laboratory methodologies, and outcome measures. This variability complicates direct comparison between studies and limits the ability to draw definitive conclusions. This variability limits reproducibility and complicates interpretation when considering both immunoglobulin-based biomarkers and therapeutic interventions such as IVIG. Therefore, more large-scale, standardized prospective studies with better patient stratification are required to identify clinically-meaningful subgroups of immune-mediated RPL.

Investigating how IgG subclasses modulate immune responses at the maternal-fetal interface is an emerging area of research [88]. Each IgG subclass differs in its potential for complement activation and Fc receptor binding, which may lead to differential effects on trophoblast survival and placental development [73]. However, current evidence remains limited, and the precise contribution of individual subclasses to RPL pathogenesis has yet to be clearly defined.

The therapeutic role of IVIG remains debated, despite evidence of benefit in selected patient populations [17]. While some studies suggest improved outcomes in women with defined immunological abnormalities, randomized controlled trials in unselected RPL populations have produced variable results. This discrepancy is likely attributable to heterogeneity in treatment protocols, dosage, timing of administration, and patient selection criteria [37]. These findings support the need for individualized, mechanism-based therapeutic approaches guided by immunological profiling.

Recent evidence suggests that, in addition to antibody levels, factors such as immunoglobulin glycosylation and Fc receptor interactions may influence immunomodulatory effects during pregnancy [89, 90]. These emerging insights highlight additional layers of complexity in immunoglobulin-mediated regulation and may provide novel targets for future diagnostic and therapeutic approaches [28].

Overall, although immunoglobulin-mediated mechanisms provide valuable insights into RPL, current evidence is insufficient to support their routine clinical application without further validation. Large-scale, well-designed prospective studies integrating immunological, clinical, and molecular data are essential to translate these findings into effective precision-based management strategies [79].

## Challenges, controversies, and knowledge gaps

Although immune-mediated mechanisms in RPL are becoming increasingly accepted, continued challenges and gaps in knowledge exist. One of the most significant hurdles is that there is no consensus on the definition of RPL across professional societies. The American Society for Reproductive Medicine (ASRM) defines RPL as two or more failed clinical pregnancies confirmed by ultrasound or histopathology, whereas the European Society of Human Reproduction and Embryology (ESHRE) defines it as two or more pregnancy losses before 24 weeks of gestation. These discrepancies in inclusion criteria complicate patient classification, study design, and cross-study comparison.

Another barrier to achieving diagnostic uniformity is the lack of universally accepted cut-off points and standardized assay platforms (e.g., ELISA) for immunological measurements, including autoantibody titers and activity of immune cells. As a result, existing data cannot be replicated or applied clinically. As such, many of the immunological markers lack the sensitivity and specificity necessary for reliable use as stand-alone diagnostic markers.

The evidence-base for using immunoglobulin therapies, particularly IVIG, is still controversial due to the lack of well-designed, adequately powered randomized controlled trials. In addition, there is a large variability in trial outcomes due to variation in treatment protocols and sample sizes and the heterogeneous nature of the sample cohorts. Furthermore, the cost of accessing IVIG, the variability of access to IVIG, and the unknown efficacy of IVIG in the unexplained subpopulations of RPL contributes to questions of the quality of care and to the inequity of health care.

A second, conceptual issue that must be confronted is the difficulty of translating the causality and correlation that takes place with respect to the continually changing and evolving dynamics of the normal pregnant immune system. Whether immunoglobulin disturbance is causative or secondary in RPL remains unclear, e.g., is the change of the immunoglobulin level causally or incidentally to the RPL?

## Future perspectives and research directions

To advance the study and management of recurrent miscarriage, it will be necessary to adopt a more standardized, mechanism-based, individualized, and personalized approach to these conditions. A major limitation is the lack of standardization associated with the cutoff values used to perform these tests, the assay platforms on which these tests are conducted, the clinical correlates associated with these test results, and the inconsistent reporting of test results represents a major obstacle to advancements in diagnosing and managing RPL. The establishment of uniformly accepted standards for the measurement of immunoglobulin antibodies that will include subclass profiles, isotypes, and longitudinal assessments of test results would enhance the quality of diagnostic testing and facilitate comparisons across studies.

Immunomodulation as therapy for RPL has been traditionally provided using intravenous immunoglobulin, but it is also being studied through alternative immunomodulatory therapies. Additionally, other immunomodulatory therapies that provide targeted approaches by modulating the complement pathways, Fc receptor signalling or specific immune cell populations may be more efficacious than traditional therapies, and will also be less expensive and less likely to cause adverse effects. Innovations in biologics and small-molecule immunomodulatory therapies now enable clinicians to selectively inhibit pathogenic immune responses while still maintaining protective immune responses.

Multi-omics technologies like transcriptomics, proteomics, immunophenotyping, and genomics will improve understanding of the complex application of immunology to RPL and provide insight into potential immunological failure through comprehensive immune profiling efforts, which will identify unique molecular signatures of immunological tolerance failure. Identifying new biomarkers and therapeutic targets. By combining immune cell phenotype and omics data, high-risk individuals can be identified sooner through improved predictive classification.

The ultimate goal of RPL management related to immune-mediated causes is patient-specific immunotherapy. Stratifying patients based on their immunoglobulin profiles, immunological signatures, and clinical characteristics enables treatment plans that are tailored for maximum benefit and minimal unnecessary interventions. Monitoring immune parameters longitudinally may provide an assessment of treatment response and lead to modifications of therapy. Collectively, the above future directions mark a transition from empirical treatment of RPL to precision medicine, providing hope for improved outcomes associated with RPL.

## Conclusion

Immunoglobulins are important regulators of the maternal-fetal immune system. They also play a key role in the pathogenesis of recurrent pregnancy loss from an immune-mediated standpoint. Immunoglobulins act as both biomarkers and mediators of immune dysfunction, supporting improved diagnostic precision and the development of targeted therapies. Although immunoglobulin-based therapies (such as IVIG) have shown promise in selected patient subsets, their routine clinical use is not currently supported by major practice guidelines. ASRM and ESHRE do not recommend routine IVIG for unexplained RPL due to insufficient high-quality evidence. Future research should focus on using biomarkers for patient stratification and clarifying immunoglobulin-mediated mechanisms to improve the clinical effectiveness of immunoglobulin-based therapies.

## Supplementary Information

Below is the link to the electronic supplementary material.

Supplementary Material 1

## Data Availability

No datasets were generated or analysed during the current study.
