# Supplementary material for: Immunoglobulins in recurrent pregnancy loss: Emerging biomarkers and therapeutic targets
Source: Mol Biol Rep. 2026 Jul 31;53(1):1317. doi: 10.1007/s11033-026-12514-2 (PMC13427805; doi:10.1007/s11033-026-12514-2)
Supplement: Supplementary file 1 — Supplementary Material 1 [file 11033_2026_12514_MOESM1_ESM.docx]

**Supplementary Material:**

**Supplementary Table 1.** Conventional etiological factors in recurrent pregnancy loss and their limitations

| **Etiological Category** | **Representative Conditions** | **Pathophysiological Basis** | **Key Limitations in Explaining RPL** |
| --- | --- | --- | --- |
| Genetic [91] | chromosomal abnormalities (e.g., aneuploidy, translocations) | Embryonic lethality due to genomic imbalance | Explains mainly sporadic losses; limited role in unexplained RPL |
| Anatomical [92] | Uterine anomalies (septate uterus, fibroids, intrauterine adhesions) | Impaired implantation and uteroplacental blood flow | Absent in the majority of RPL cases |
| Endocrine [93] | Thyroid dysfunction, PCOS, luteal phase defects | Hormonal imbalance affecting implantation and early development | Partial contribution; often correctable but not universally causative |
| Infectious [94] | TORCH infections, chronic endometritis | Inflammatory damage to endometrium and placenta | Rare cause of recurrent (as opposed to sporadic) losses |
| Thrombotic [95] | Antiphospholipid syndrome (APS), inherited thrombophilia | Placental thrombosis and vascular insufficiency | Accounts for only a subset of patients |
